# ASSESSING HEALTH BEHAVIORS AND WELLBEING OF NON-SPOUSAL LGBTQIA+ CAREGIVERS OF PEOPLE WITH AD/ADRD

**DOI:** 10.1093/geroni/igae098.4119

**Published:** 2024-12-31

**Authors:** Clover Caldwell, Heather Schwartz, A J Slowey, Nicole Werner, Andrew Pickett

**Affiliations:** Indiana University Bloomington, Bloomington, Indiana, United States; Indiana University Bloomington, Bloomington, Indiana, United States; Indiana University Bloomington, Bloomington, Indiana, United States; Indiana University Bloomington, Bloomington, Indiana, United States; Indiana University Bloomington, Bloomington, Indiana, United States

## Abstract

Informal caregivers of people living with Alzheimer’s Disease (AD) or AD-Related Dementias (AD/ADRD) face high levels of burden and stress, while LGBTQIA+ identifying individuals often experience stigmatization and discrimination. Young adults are increasingly involved in caregiving for older adult relatives. To date, there is limited research related to the experiences LGBTQIA+ dementia caregivers, particularly those who are not spousal partners of care recipients. We conducted an online survey of LGBTQIA+, non-spousal caregivers, recruited through the Prolific platform. Participants (n=116) responded to items about health-behaviors (e.g., physical activity, sleep hygiene, alcohol use) and caregiving experiences (e.g., burden). Participants most commonly identified as white (n=63, 53.4%), women (n=64, 54.2%), and bisexual (n=71, 60.2%). The average age was 37.64 (SD=12.44) years and participants reported 25.54 (SD=25.17) average weekly hours caring for parents (n=56, 48.3%), grandparents (n=40, 34.5%), or others (n=24, 20.7%). Participants most commonly were ‘moderately active’ (n=70, 60.3%), had poor sleep quality (M=2.32 / 4, SD=.87), moderate alcohol use (n=52, 50.5% reported 3-6 drinks per session) and caregiver burden (M=13.54, SD=7.19). Participants reported feelings of loneliness (M=5.39/ 9, SD=2.04) and depressive symptoms (M=9.32, SD= 7.22), both of which were predicted by caregiver burden (Loneliness: F=2.73, p<.001, Adj. R2=.30; Depressive Symptoms: F=2.92, p<.001, Adj. R2=.32). Non-spousal LGBTQIA+ dementia caregivers reported suboptimal health behaviors, partially associated with time and other constraints related to caregiving. Further, caregiver burden was associated with both loneliness and depressive symptoms. Additional research and interventions are needed to promote health and reduce burden among this population.

